# ASO Author Reflections: Sentinel Lymph Node Biopsy for Patients with a Non-classic Lobular Carcinoma In Situ Breast Biopsy

**DOI:** 10.1245/s10434-024-16863-9

**Published:** 2025-01-17

**Authors:** Pieter J. Westenend, Claudia J. C. Meurs, Sabine Siesling, Marian B. E. Menke-Pluijmers

**Affiliations:** 1 Laboratory of Pathology Dordrecht, Dordrecht, The Netherlands; 2 Department of Health Technology and Services Research, Technical Medical Centre, University of Twente, Enschede, The Netherlands; 3 CMAnalyzing, Zevenaar, The Netherlands; 4 Department of Research, Netherlands Comprehensive Cancer Organisation, Utrecht, The Netherlands; 5 Department of Surgery, Albert Schweitzer Hospital, Dordrecht, The Netherlands

As a primary diagnosis, pleomorphic or florid lobular carcinoma *in situ* (LCIS), also known as non-classic LCIS, is a rare finding on breast biopsy. A population-based study in the Netherlands found 160 cases during 10 years.^1^ At resection, 31–32 % of the patients were upstaged to invasive carcinoma.

Based on expert opinion and consensus, guidelines recommend that these patients should be treated similarly to patients with a ductal carcinoma *in situ* (DCIS) biopsy (i.e., by surgery). Like many other guidelines, the Dutch guideline does not specify whether this means that performing a sentinel lymph node biopsy (SLNB) in selected cases is part of this recommendation.^2^ According to this guideline, SLNB should be considered for patients with DCIS biopsy at high risk for upstaging who are scheduled for breast-conserving surgery (BCS). We found that SLNB was performed for 48 (36 %) of the 133 surgically treated patients with a non-classic LCIS biopsy. This is lower than the SLNB rate of 67 % for patients with a DCIS biopsy.^3^

The role of an SLNB for patients with a DCIS biopsy is the subject of an ongoing debate. In a recent guideline, the European Society of Breast Cancer Specialists (EUSOMA) set the SLNB target for patients treated with breast-conserving surgery (BCS) at 10 %, with a maximum of 20 %.^4^ In our LCIS study, we found an SLNB rate of 28 % (30 of 106) for patients undergoing BCS and 67 % (18 of 27) for patients undergoing mastectomy (unpublished analysis).^1^.

Selection of patients based on risk factors for upstaging is critical to lowering the SLNB rate. Unfortunately, because non-classic LCIS is so rare, there are no data on risk factors for upstaging in non-classic LCIS. On the other hand, it is reassuring that we found a metastasis rate of 6 % (2 patients with a micro-metastasis and 1 patient with a macro-metastasis, excluding 3 patients with isolated tumour cells)^1^. For patients treated by BCS, we found only one micro-metastasis.

The recently published SOUND trial demonstrated no difference in distant metastasis, survival, or local recurrence between patients with small (<2 cm), clinically node-negative invasive cancers treated with an SLNB and those treated without an SLNB.^5^ The metastasis rate for patients treated with an SLNB was 13.7 %, twice as high as in our LCIS study. In our study, 34 patients had invasive breast cancer at surgery, 4 of whom had a tumor larger than 2 cm.

In our opinion, this means that the SLNB target rate for non-classic LCIS can be the same 10 % as for DCIS, and that not performing a SLNB is also an option. We encourage researchers studying non-classic LCIS to report not only on the upstage rate, but also on the use and outcomes of SLNB because the data are sparse.
